# Cross-cultural adaptation and psychometric testing of the Turkish Version of the Workplace Activity Limitations Scale (WALS) in people with inflammatory arthritis

**DOI:** 10.1093/rap/rkag028

**Published:** 2026-02-27

**Authors:** Gonca Bumin, Ezginur Gündoğmuş, Alan Tennant, Sevilay Karahan, Umut Kalyoncu, Yeliz Prior

**Affiliations:** Department of Occupational Therapy, Faculty of Health Sciences, Hacettepe University, Ankara, Turkey; Department of Occupational Therapy, Faculty of Health Sciences, Hacettepe University, Ankara, Turkey; Leeds Institute of Rheumatic and Musculoskeletal Medicine, University of Leeds, Leeds, UK; Department of Biostatistics, Faculty of Medicine, Hacettepe University, Ankara, Turkey; Department of Rheumatology, Faculty of Medicine, Hacettepe University, Ankara, Turkey; Centre for Human Movement and Rehabilitation, School of Health and Society, University of Salford, Salford, UK; Versus Arthritis/MRC Centre for Musculoskeletal Health and Work, School of Health and Society, University of Salford, Salford, UK

**Keywords:** rheumatologic diseases, inflammatory arthritis, work, vocational rehabilitation, patient-reported outcomes, psychometry

## Abstract

**Objectives:**

The Workplace Activity Limitations Scale (WALS) is a widely used measure to assess work-related functional limitations in individuals with rheumatologic diseases. However, no validated Turkish version of the WALS exists, limiting its applicability in Turkish-speaking populations. This study aims to conduct a cross-cultural adaptation and psychometric evaluation of the Turkish version of WALS in individuals with inflammatory arthritis (IA).

**Methods:**

WALS was forward and backward translated, and cognitive debriefing interviews were conducted with 30 participants to assess comprehensibility and cultural relevance. Then, 200 participants completed the Turkish version of the WALS along with the Work Limitation Questionnaire-Short Form (WLQ-SF), the Rheumatoid Arthritis Work Instability Scale (RA-WIS), the Work Productivity Activity Impairment General Health V2.0 (WPAI-GH), the Health Assessment Questionnaire (HAQ) and the Rheumatoid Arthritis Impact of Disease (RAID). Of these, 79 participants also completed the WALS again 2 weeks later to assess test–retest reliability.

**Results:**

WALS showed high internal consistency (Cronbach’s alpha = 0.89) and excellent test–retest reliability (ICC (2.1): 0.91). WALS met the Rasch model requirements for fit. Concurrent validity showed that the WALS total score was moderately correlated with the WLQ total score (*r* = −0.57), some parameters of the WPAI (*r*^2^ = 0.42; *r*^3^ = 0.40; *r*^4^ = 0.43), HAQ (*r* = 0.54) and RAID (*r* = 0.57).

**Conclusion:**

The Turkish version of the WALS is a valid and reliable instrument for assessing workplace activity limitations in individuals with IA. It can be used in clinical and research settings to assess work-related functional difficulties and guide occupational interventions for Turkish-speaking individuals with IA.

Key messages

- The Turkish WALS works well and gives consistent results for people with inflammatory arthritis.
- It accurately reflects the kinds of difficulties people experience with work.
- It can be used in healthcare and research to help identify work-related challenges and support people to stay in work.

## Introduction

Inflammatory arthritis (IA) encompasses chronic, progressive autoimmune conditions affecting joints, such as rheumatoid arthritis (RA), psoriatic arthritis (PsA), axial spondyloarthritis (axSpA) and systemic lupus erythematosus (SLE), leading to significant physical and functional limitations [1–3]. The symptoms of these diagnostic groups are unique; however, joint pain, deformity and systemic symptoms in the pathway provide a unique form of commonality [4]. The presence of these symptoms decreases workplace activity performance and work productivity in individuals with IA [5]. Such symptoms contribute to physical limitations in patients and prevent adaptation to the daily working pace by reducing the required capacity to fulfil work demands and activities that require fine and/or gross manual dexterity and endurance [6, 7]. These conditions can also result in pain and fatigue, leading to cognitive effects, including brain fog or trouble focusing [8]. All these conditions have been reported to impair the capacity of individuals to meet job task performance and to increase the prevalence of absenteeism, decrease capacity for work and furthermore lead, at worst, to job loss [6, 7, 9, 10].

Measurement tools used to assess workplace and work limitations are critical to guide rehabilitation efforts and support individuals to maintain employment. The Workplace Activity Limitation Scale (WALS) is an assessment tool that aims to provide an objective measure of clinical evaluations of workplace limitations, the degree of difficulty for individuals and workplace adaptations [11–13]. WALS was developed in Canada [11], and validity and reliability studies were conducted in the United Kingdom (UK) [13], and it was reported to have good psychometric properties. The WALS assesses the degree of difficulty in performing specific work activities and identifies limitations. It is frequently used to assess workplace difficulties in individuals with IA [13, 14] and osteoarthritis (OA) [15], as well as SLE [16] and systemic sclerosis (SS) [15]. Other assessment tools developed to assess work productivity and limitations in people with IA include the Work Limitations Questionnaire (WLQ-25) [17] and the Turkish version of the Work Limitations Questionnaire Short Form (WLQ-Short Form) [18] which assess personal (physical and emotional) limitations and how they may affect the ability to perform work.

However, these tools mainly focus on productivity loss or generalized disability rather than the specific activity limitations encountered in the workplace. In contrast, the WALS was specifically designed to assess the degree of difficulty in performing specific work tasks, including physical, cognitive and environmental aspects of functioning [13, 19]. It is a concise, patient-reported outcome measure that aligns with the International Classification of Functioning, Disability and Health (ICF) framework and provides clinically useful data to guide occupational therapy and vocational interventions [16, 20]. Given these features, the WALS offers a distinct advantage over broader instruments such as the WLQ, and its cross-cultural adaptation into Turkish fills an important gap in the availability of function-specific workplace assessment tools. To date, the WALS has not been culturally adapted or psychometrically validated for use with Turkish-speaking populations. This study aimed to translate and culturally adapt the WALS into Turkish and to evaluate its psychometric properties in individuals with IA experiencing work-related limitations.

## Methods

### Participants

This study included 200 individuals diagnosed with IA by a rheumatologist at the Hacettepe University Faculty of Medicine, Department of Rheumatology, Ankara, Turkiye. Eligibility criteria were: age ≥18 years and current paid employment (full- or part-time, for at least one day per week). Participants on long-term sick leave or with prolonged illness were excluded, as the presenteeism-related measures required respondents to reflect on their work ability over the past 2 weeks. Ethical approval was granted by the Hacettepe University Health Sciences Research Ethics Committee (Decision No: 2024/01-03), and written informed consent was obtained from all participants.

### Sample size

A minimum of 150 participants is recommended for Rasch analysis to adequately assess construct validity [21]. To enhance the representativeness and variability of responses, we targeted a sample size of 200 individuals. For test–retest reliability, it was calculated that at least 79 participants were needed to detect a correlation of 0.7 as statistically different from a background correlation of 0.45, with 90% power at a 1% significance level. A correlation coefficient of 0.7 was considered acceptable for establishing test-retest reliability [22].

### Study design

Permission to adapt the WALS was obtained from Prof. Gignac, the original developer of the scale. The cultural adaptation followed the standardized translation methodology outlined by Beaton *et al.* [23]. In addition, the study adhered to the COSMIN (Consensus-based Standards for the Selection of Health Measurement Instruments) guidelines to ensure rigorous evaluation of measurement properties [24].

### Data collection

#### Phase 1: Linguistic and cultural validation

The translation, linguistic validation, and cross-cultural adaptation of the Turkish WALS are detailed in Supplementary File 1, available at *Rheumatology Advances in Practice* online. During cognitive debriefing interviews with 30 participants, all items were deemed highly relevant by participants. Based on feedback from the expert panel, only minor wording adjustments were made to enhance cultural appropriateness. In item 7, the word ‘reaching’ was translated as ‘uzanmak’ in the forward translation. However, since ‘uzanmak’ was translated as ‘lying down’ in the backward translation, it was changed to ‘ulaşmak’ to accurately reflect the meaning.

#### Phase 2: Implementation of psychometric tests

Participants completed the Turkish WALS along with several other validated work and health-related questionnaires to assess concurrent validity by comparing the WALS against measures of similar and related constructs.

Demographic data were collected on age, height, weight, BMI, sex, diagnosis, duration of diagnosis and symptoms, employment status, work-related conditions, education level, marital status and current medications.

### Workplace Activities Limitations Scale (WALS)

The WALS measures the level of difficulty experienced in performing work-related tasks [19]. Items address challenges such as commuting, moving around the workplace, prolonged sitting or standing, lifting, hand use, squatting, bending or kneeling, reaching, work schedule, pace and meeting job demands. Responses are rated on a 4-point Likert scale (0 = no difficulty; 1 = some difficulty; 2 = a lot of difficulty; 3 = unable to do). Scores of 0–6 indicate low work instability, 7–13 moderate instability and 14–36 high instability [24]. The scale has demonstrated strong reliability in individuals with RA (Cronbach’s α = 0.87; median score 9.00, IQR 5.00–14.00; range 0–36) [13].

### Scales used to test concurrent validity

To evaluate concurrent validity, participants also completed the WLQ-SF, the Rheumatoid Arthritis Work Instability Scale (RA-WIS), the Work Productivity and Activity Impairment General Health Version 2.0 (WPAI: GH), the Health Assessment Questionnaire (HAQ) and the Rheumatoid Arthritis Impact of Disease (RAID) scale. All these instruments have validated Turkish versions WLQ-SF [18], WPAI-GH [25], HAQ [26] and RAID [27], which were used in the present study. Additionally, several health-related variables were assessed using numeric rating scales (NRS). Participants were asked to rate their confidence in working (0 = not confident at all, 10 = completely confident), mood (0 = very poor, 10 = very good) and severity of hand pain (0 = no pain, 10 = worst possible pain). Overall health status was evaluated on a 5-point NRS (1 = very poor, 5 = very good). The physical demands of the job were measured using a self-report single-item question asking participants to classify the overall physical intensity of their work as low, moderate or high. Participants selected the category that best described their job demands based on their daily work activities.

### Test–retest reliability

To assess test–retest reliability, the first 79 participants were invited to complete the Turkish WALS a second time, 2 weeks after the initial administration. The s.e.m. and minimal detectable change (MDC_95_) were calculated based on test–retest reliability data to estimate measurement precision.

### Statistical analysis

All analyses were performed with IBM SPSS version 23.0 package program. Numerical variables were summarized as mean ± s.d., median (25th–75th percentile) and categorical variables were summarized as number and percentage. Graphical analysis and Kolmogorov–Smirnov test were used to assess the conformity of numerical variables to normal distribution. The scale’s internal construct validity was evaluated using Rasch analysis [28]. The scale was tested against the requirements of the Rasch Measurement Model using the partial credit parametrization [28, 29]. A *P-value* < 0.05 was accepted for statistical significance.

The analysis was undertaken in a calibration sample of 200 cases taken from two time points where no individual was included more than once [30]. Invariance was tested through Differential Item Functioning (DIF) of contextual factors, which included time, age, sex, diagnosis, medication, Body Mass Index (BMI) and duration of symptoms [21]. Cross-cultural invariance was tested by comparison with the data from the English version. Monotonicity was tested by threshold ordering, and homogeneity through examination of the probabilistic ordering of items with Chi-Square fit statistics. Local Item Independence was tested through correlations within the item residuals [31]. Where this test failed, consideration was given to grouping locally dependent items into ‘super-items’ by simply adding them together [32]. If a conceptually based grouping is also possible, then ‘testlets’ can be derived and, if the analysis results in two ‘testlets’ or ‘super-items’, a conditional Chi-Square fit statistic becomes available [33]. Unidimensionality is tested through Smith’s *t*-test of residuals [34].

Should fit be achieved, then the result is tested for invariance (by DIF) against the British English version [13]. Should invariance be achieved then comparison between countries will be valid. Else, information may be gained about where such lack of invariance is found, providing information back to the developers.

Factor analysis identified the domains measured by the item sets [35]. The Person Separation Index, a measure of internal consistency, was derived from Rasch analysis. This is equivalent to Cronbach’s alpha and was used to report the ability of the scales to discriminate between respondents.

#### Content validity

The scales were checked against the ICF Core Set for Vocational Rehabilitation [36] using ICF linkage rules. Two researchers independently correlated the items and reached a consensus to identify the ICF domain items (Supplementary File 2, available at *Rheumatology Advances in Practice* online).

#### Concurrent validity

To demonstrate concurrent validity, the relationship between the work questionnaires WLQ-SF, RA-WIS and WPAI-GH, and the health questionnaires HAQ and RAID was determined by Spearman correlation coefficient (rho). Correlation coefficients were interpreted according to Cohen’s classification: values around 0.10–0.29 were considered low, 0.30–0.49 were considered moderate, and 0.50 and above were considered strong correlations [37].

#### Reliability

Intraclass correlation coefficient (ICC) and the Wilcoxon test were applied to demonstrate the test–retest reliability of WALS. Internal consistency was tested by calculating Cronbach’s *α*. An ICC value of ≥0.75 and a Cronbach’s *α* value of ≥0.70 were considered desirable [38]. Corrected item-total correlations and item-deleted Cronbach’s *α* values were also evaluated for each subscale. Corrected item-total correlations higher than 0.30 were accepted [22].

#### Floor and ceiling effects

A floor and ceiling effect of less than 15% indicates that the scale does not cluster at extreme values and is able to measure sensitively across a wide range of individuals [39].

## Results

Cognitive debriefing interviews (*n* = 30) confirmed that all items were considered highly relevant. The participants in this phase had a mean age of 43.0 ± 12.3 years (range 23–64), and 73.3% were female. Following review by the expert panel, no further wording modifications were necessary, and the final version of the Turkish WALS was confirmed. Demographic characteristics of participants are presented in Table 1. No significant differences in age were found across medication types (*F* = 0.12; df = 3196; *P* = 0.948), nor between sexes (chi-square = 2.458; df = 3; *P* = 0.483).

**Table 1:** Demographic information of Phase 1 and Phase 2 participants.

		Phase 1		Phase 2	
		Mean ± s.d.	Min–Max	Mean ± s.d.	Min–Max
Age		43.0 ± 12.3	23–64	37.9 ± 10.6	19–66
Height		160.7 ± 31.2	152–188	168.6 ± 8.7	145–190
Weight		72.6 ± 13.0	53–105	70.3 ± 12.0	48–105
BMI		26.4 ± 4.5	20.9–39.1	24.7 ± 3.7	18.0–39.1
Duration of diagnosis		11.4 ± 10.7	1–35	9.2 ± 8.0	1–35
Duration of complaints		12.7 ± 10.6	1–36	10.6 ± 8.3	1–36
Average working h per week		39.1 ± 11.7	8–60	42.3 ± 9.3	10–72
Confidence in working		6.56 ± 3.4	0–10	7.3 ± 2.9	0–10
Mood		4.7 ± 2.7	0–10	4.6 ± 2.7	0–10
Overall health status		3.0 ± 0.8	1–5	2.9 ± 0.8	1–5
Severity of hand pain		3.6 ± 3.1	0–10	4.3 ± 3.2	0–10
		**Frequency**	**Percent**	**Frequency**	**Percent**
Sex	Female	22	73.3	133	66.5
	Male	8	26.7	67	33.5
Diagnosis	Rheumatoid arthritis	21	70	130	65
	Axial spondyloarthritis	9	30	49	24.5
	Spondylarthritis			1	0.5
	Psoriatic arthritis			20	10.0
Workplace size	0–50 person	15	50	75	37.5
	50–100 person	3	10	40	20.0
	100+	12	40	85	42.5
Employment status	Full time	27	90	187	93.5
	Part-time	3	10	13	6.5
Physical demands of the job	Low	10	33.3	45	22.5
	Moderate	15	50	96	48.0
	High	5	16.7	59	29.5
Education level	Primary school	1	3.3	7	3.5
	Secondary school	2	6.7	7	3.5
	High school	5	16.7	23	11.5
	University	19	63.3	129	64.5
	Postgraduate	3	10	34	17.0
Marriage status	Partnered	19	63.3	134	67.0
	Single	11	36.7	66	33.0
Current medication type	None			6	3.0
	NSAID	1	3.3	6	3.0
	Steroid			9	4.5
	Single DMARD	20	66.7	101	50.5
	Combination DMARD	4	13.3	34	17.0
	Neuropathic analgesic			1	0.5
	Biologic/biosimilar	1	3.3	15	7.5
	Steroids, NSAIDs			1	0.5
	NSAIDs, analgesic	4	13.3	27	13.5

DMARD: disease-modifying antirheumatic drugs; NSAID: non-steroidal anti-inflammatory drug; s.d.: standard deviation.

The work and health assessment results for the 200 participants are presented in Table 2.

**Table 2:** Descriptive statistics for the Phase 2 participants’ work and health measures scores (*n* = 200).

	Mean ± s.d.	Median [25th–75th percentile]
WALS total	11.47 ± 5.57	12 [7–16]
WLQ-TM	3.16 ± 1.16	3 [2.5–4]
WLQ-PD	2.45 ± 0.89	2.5 [2–3]
WLQ-MID	3.77 ± 0.87	4 [3–4.5]
WLQ-OD	3.52 ± 1.12	3.5 [3–4.5]
WLQTOTAL	12.90 ± 2.39	13 [11.5–14.5]
WLQpercentageloss	15.04 ± 4.44	14.58 [11.85–18.58]
RAWIS	12.04 ± 6.40	13 [7–17]
WPAI: GH1	13.14 ± 22.53	0 [0–18]
WPAI: GH2	44.00 ± 25.12	50 [20–60]
WPAI: GH3	34.92 ± 21.65	30 [20–50]
WPAI: GH4	48.80 ± 26.11	50 [30–70]
HAQ	13.27 ± 4.05	13 [10–17]
RAID	4.95 ± 2.23	4.8 [2.94–6.96]

HAQ: Health Assessment Questionnaire; MID: mental-interpersonal demands; OD: output demands; PD: physical demands; RAID: Rheumatoid Arthritis Impact of Disease L; RAWIS: Rheumatoid Arthritis Work Instability Scale; TM: time management; WALS: Work Assessment Limitation Scale; WLQ: Work Limitation Questionnaire; WPAI: Work Productivity Activity Impairment.

### Validity

#### Construct (structural) validity

Fit of the WALS to the Rasch model is shown in Table 3. All items displayed ordered thresholds. While fit and reliability were acceptable, the initial item-based model was not satisfactory, with multidimensionality derived from two groups of items relating to access to work and managing h such as ‘managing your current job demands’ (designated Environment), and the physical aspects such as ‘Lifting, Carrying or moving objects’ (designated Physical Functioning). One pair of items also showed a lack of acceptable item independence. The items ‘Managing the shifts of h of work that your job requires’ and ‘Managing the pace of work that your job requires’ had a residual correlation of 0.343 with an average residual correlation in the item set of −0.088. Grouping these two items into a ‘super item’ did not improve fit. Thereafter, a bi-factor ‘testlet’ based solution (conceptually based with environment and physical functioning item sets) resolved both fit and dimensionality, retaining 91% of the variance of the data.

**Table 3:** Fit of WALS to the Rasch model.

Analysis type	Residuals		Test of fit			Reliability		Dimensionality		DIF	Variance
	Item	Person	χ	df	*P*	α	PSI	*t*-test	LCI		
Turkish											
Item-based	1.713	1.413	34.6	24	0.075	0.89	0.88	10.0	7.0	Time	–
Super item	1.494	1.311	26.6	20	0.148	0.87	0.87	7.5	4.5	Time	0.97
Testlet	0.862	0.921	32.4	18	0.020	0.81	0.82	2.5	–	Time/Sex	0.91
Turkish-English	2.249	0.972	29.7	21	0.099	0.78	0.77	4.1	–	Environment	0.89
Ideal value	**<1.4**	**<1.4**			**>0.017**	**>0.7**	**>0.7**	**<5.0**	**<5.0**		**>0.9**

*α*, Cronbach’s alpha; DIF, differential item functioning; LCI, lower confidence interval; PSI, Person Separation Index.

All contextual factors showed invariance except Time, where one item, ‘As a result of your arthritis, how much difficulty do you have concentrating or keeping your mind on your work?’ showed a significant difference across time, where those responding to the retest (time 1) showed higher values for this item. This item was split into two separate time variables, and the person estimates compared from the unsplit and split solutions (the latter anchored on the 11 item difficulties from the unsplit solution), showing no significant difference between estimates (*t*-test *P* = 0.855). Consequently, the unsplit solution was retained. Cross-cultural invariance, tested against the original English data, showed adequate fit to the Rasch model, retaining 89% of the variance and strict unidimensionality with the testlet-based version. While country DIF was evident with the ‘environment’ testlet, the difference between estimates when the testlet was split gave a paired-test of 0.291 so no further action was taken.

Further information on the Rasch analysis is given in Supplementary File 3, available at *Rheumatology Advances in Practice* online.

##### Work profile

No association was found between work limitations and the duration since diagnosis (*F* 1.4 df 3196; *P* = 0.244). Neither was with Body Mass Index (BMI) (*F* 186 df 3196; *P* = 0.139). There was a significant difference in the average number of h worked each week by diagnostic group. Those with RA had an average working h of 25.4 (s.d. 21.7), compared with, say, axSpA at 41.2 h (s.d. 8.6) (*F* 8.9, df 2197; *P* =< 0.001). However, there was no significant difference in the level of limitations by diagnostic group (*F* 0.79 df 2197; *P* = 0.456). Neither was there any significant difference across the reported physical demand of their job (*F* 1.37 df 2197; *P* = 0.690). Just over half (51.5%) were educated to degree level and above, and there was no significant relationship between work limitations and education level (*t* 1.59, df 198; *P* = 0.113).

Half worked in establishments with 0–50 employees, whereas almost a quarter (23.5%) worked in establishments with over 100 employees. There was a significant gradient of work limitations across the size of the establishment, with those in small establishments (0–50) having a higher metric WALS score, indicating higher limitations (*F* 4.15; df 2197; *P* = 0.017). The effect size for the difference in WALS between the smallest and largest establishment was 0.52, considered medium. There was a strong association between reported confidence in working (grouped low-medium-high from a 0–10 NRS) with size of establishment (chi-square 71.4 (df 4); *P* =< 0.001). The smaller the workplace size, the lower the confidence in working. Limitations in work also reflected confidence in work, with higher limitations for those with low confidence (*F* 8.71 df 2197; *P* =< 0.001). The effect size of the difference in WALS between those with low and high confidence was 0.58.

#### Concurrent validity

The correlation values of the scale total score with other scales are given in Table 4. When the correlations of the total score of the scale with other scales were examined, it was seen that there was a negative moderate correlation with the dimensions of the WLQ, except for PD and the total score. The correlation with RAWIS was not statistically significant. It has a weak positive correlation with WPAI-1 and a moderate positive correlation with WPAI-2, 3 and 4 scores. It has a moderate positive correlation with HAQ and RAID. The total score of the scale has a negative moderate correlation with the question ‘confidence in working’. It has a moderate positive correlation with the questions ‘mood, overall health status and severity of hand pain’ (Table 4).

**Table 4:** Concurrent validity of the WALS with work and health measures.

	***ρ*** (rho)
WLQ-TM	−0.564**
WLQ-PD	0.449**
WLQ-MID	−0.564**
WLQ-OD	−0.573**
WLQTOTAL	−0.574**
WLQ percentage work loss	−0.629**
RAWIS	−0.103
WPAI: GH1	0.117
WPAI: GH2	0.424**
WPAI: GH3	0.400**
WPAI: GH4	0.430**
HAQ	0.546**
RAID	0.573**
Confidence in working NRS (0–10)	−0.374**
Mood NRS (0–10)	0.364**
Overall health status NRS (1–5)	0.232**
Severity of hand pain NRS (0–10)	0.362**

HAQ: Health Assessment Questionnaire; MID: mental-interpersonal demands; NRS: Numeric Rating Scale; OP: output demands; PD: physical demands; RAID: Rheumatoid Arthritis Impact of Disease; RAWIS: Rheumatoid Arthritis Work Instability Scale; TM: time management; WPAI: Work Productivity Activity Impairment; WLQ: Work Limitation Questionnaire.

** *P* < 0.0001.

Item–total correlations ranged between 0.482 and 0.667. The internal consistency coefficient of the scale is 0.883. When the item was deleted, Cronbach’s alpha values were similar for all items (Table 5).

**Table 5:** Item statistic of WALS.

	Mean	s.d.	Corrected item-total correlation	Cronbach’s alpha if item deleted
WALS1	0.785	0.68639	0.666	0.869
WALS2	0.965	0.69004	0.621	0.872
WALS3	0.875	0.70131	0.536	0.877
WALS4	1.21	0.76079	0.542	0.877
WALS5	1.235	0.68712	0.566	0.875
WALS6	0.9	0.77004	0.499	0.880
WALS7	1.42	0.71143	0.541	0.877
WALS8	0.805	0.71381	0.662	0.869
WALS9	0.835	0.69295	0.667	0.869
WALS10	0.935	0.65798	0.617	0.872
WALS11	0.75	0.65548	0.482	0.880
WALS12	0.76	0.65155	0.607	0.873

s.d.: standard deviation; WALS: Work Assessment Limitation Scale.

### Test–retest reliability

The results of the test–retest reliability of the scale are given in Table 6. There was no statistically significant difference between the first and second test of the scale (*P* > 0.05). The test–retest relationship between the two applications was high. MDC was obtained as 3.09.

**Table 6:** Test-retest reliability.

	T1	T2	*P* (Wilcoxon test)	*ρ* (rho)	ICC(2, 1) (95% CI)	s.e.m.	MDC
WALS total	13 [9–17]	13 [9–17]	0.911	0.892**	0.913 (0.867–0.943)	1.243	3.090

ICC: intraclass correlation coefficient; MDC: minimal detectable change; s.e.m.: standard error of measurement.

** *P* < 0.001.

#### Floor and ceiling effect

Floor and ceiling effects were minimal, with 2.5% of participants scoring at the lower limit and 0.5% at the upper limit of the scale. These values indicate that the Turkish WALS did not exhibit significant floor or ceiling effects.

## Discussion

The findings of this study demonstrate that the Turkish WALS is a reliable and valid instrument for assessing work-related activity limitations in individuals with IA. These results align with previous studies [11, 13] and reinforce the value of the WALS as a key measure of functional limitations in the workplace. Moreover, the Turkish version showed good fit to the Rasch model, along with strong reliability, measurement precision, and cross-cultural equivalence with the original English version.

Construct validity was assessed through Rasch analysis, which confirmed that all items demonstrated ordered thresholds. However, multidimensionality was observed among items related to physical functioning, such as managing work demands and lifting objects. This finding is consistent with previous research, which suggests that functioning measures may reflect multiple dimensions due to the influence of physical and environmental factors [35]. We interpret this as an indication that workplace strain arises not only from individual capacity but also from physical, organizational and social demands.

Another key finding from this study was the evidence supporting the concurrent validity of the Turkish WALS. Correlation analyses showed that WALS total scores were significantly associated with other functioning-related measures, including the WLQ and WPAI. Moderate correlations were observed with both the physical and mental interpersonal demands of the WLQ, indicating a clear relationship between job demands and work limitations [40]. Similarly, moderate positive correlations with WPAI scores suggest that individuals with IA experience reduced work productivity due to disease-related limitations [41].

A moderate correlation was also found between physical activity limitations in individuals with RA and the extent to which they are affected by their condition—a relevant finding given the high proportion of participants with RA in our sample. These results reinforce the link between physical and personal impacts of rheumatological conditions and work limitations.

Additionally, WALS scores were moderately associated with general health status, severity of hand pain and mood, while showing a negative correlation with confidence in working. This suggests that functional limitations in IA are influenced not only by physical impairments but also by psychosocial factors such as self-efficacy and emotional wellbeing [42, 43]. This is supported by previous studies demonstrating that confidence in the workplace is strongly associated with labour force participation in individuals with arthritis [44, 45]. These findings underscore the importance of holistic assessment approaches that consider both physical and psychosocial dimensions when evaluating work functioning in IA. In our study, interestingly, the expected significant correlation between WALS and RA-WIS was not observed. This can be explained by the distinct constructs each scale is designed to measure. The WALS evaluates current functional limitations in workplace activities [46], whereas the RA-WIS is a prognostic tool that assesses the risk of future work instability or job loss [47]. The literature indicates that while RA-WIS focuses on perceived vulnerability to job retention, WALS captures the physical and environmental difficulties experienced during work tasks [13, 19]. Hence, the lack of correlation may reflect that individuals can experience substantial work limitations without perceiving high job instability. In our sample, many participants were employed in the public sector or in secure job environments, which may have resulted in lower RA-WIS scores despite existing physical limitations. Previous research has shown that workplace support, job security, and job-role flexibility significantly influence RA-WIS scores [14, 47]. These findings suggest that WALS and RA-WIS capture complementary but not interchangeable dimensions of work participation, reinforcing the value of using both tools in parallel for a more holistic evaluation.

The Turkish WALS demonstrated strong test–retest reliability, with an intraclass correlation coefficient (ICC) of 0.913, indicating excellent measurement stability over time. This aligns with previously reported values for the English version of the WALS, where ICC scores ranged from 0.90 to 0.94 [13]. The minimum detectable change (MDC) was calculated at 3.09, comparable to the MDC of 3.17 reported for individuals with RA in the English version, further supporting cross-cultural consistency [13]. These findings confirm that the WALS yields reproducible results in both research and clinical settings and is consistent with the ICF Vocational Rehabilitation Core Set framework [16, 48].

The standard error of measurement (SEM) was 1.243, indicating a low level of random measurement error. A low SEM suggests that the scale provides accurate estimates of true scores, enhancing its utility in individual assessments [49]. When considered alongside the MDC, this low SEM supports the WALS as a sensitive tool capable of detecting meaningful clinical changes [50]. Additionally, the SEM range for the English version (1.15–1.87) aligns closely with the result in this study [13].

No significant floor or ceiling effects were identified (2.5% and 0.5%, respectively), indicating that the scale captures a broad range of performance levels without clustering at the extremes. This wide score distribution supports its suitability for use across diverse populations and levels of work limitation.

These findings demonstrate that the Turkish WALS is a valid and reliable instrument for assessing work limitations in individuals with IA. Its application in clinical practice and vocational rehabilitation planning could support the identification of appropriate workplace accommodations and occupational therapy interventions.

### Strength and limitations

This study has several strengths, including a large and diverse sample of individuals with IA, representing a range of age groups, diagnoses, and employment contexts, which enhances the generalizability of the findings. The rigorous use of Rasch analysis also strengthens evidence for the scale’s construct validity.

However, the study is not without limitations. All participants were recruited from a single tertiary care centre, which may limit the representativeness of the wider Turkish IA population, particularly those in rural or underserved settings. Additionally, subgroup analyses by condition were limited. Future studies should examine the performance of the Turkish WALS across different diagnostic groups and work environments and evaluate its responsiveness to change over time or following workplace interventions. A limitation of this study is that responsiveness (i.e. the WALS’s ability to detect clinically meaningful changes over time) was not directly evaluated. Future longitudinal studies are needed to determine whether the Turkish version of WALS can effectively capture changes following clinical interventions.

## Conclusion

This study provides strong evidence that the Turkish version of the WALS is a valid and reliable tool for assessing workplace activity limitations in individuals with IA. The scale showed robust psychometric properties, consistent with previous validations in other languages, supporting its use in clinical practice and research settings within Turkish-speaking populations.

Further research is recommended to evaluate the scale’s performance across different regions, diagnostic groups and employment settings, as well as its responsiveness to change following vocational or therapeutic interventions.

## Supplementary Material

rkag028_Supplementary_Data

## Data Availability

The data underlying this article will be shared on reasonable request to the corresponding author.
