# Supplementary material for: Cross-cultural adaptation and psychometric testing of the Turkish Version of the Workplace Activity Limitations Scale (WALS) in people with inflammatory arthritis
Source: Rheumatol Adv Pract. 2026 Feb 27;10(1):rkag028. doi: 10.1093/rap/rkag028 (PMC12975718; doi:10.1093/rap/rkag028)
Supplement: rkag028_Supplementary_Data [file rkag028_supplementary_data.zip › Supplementary File 1.docx]

Supplementary File 1

*Phase 1: The translation, linguistic validation and cross cultural adaptation*

Two separate forward translations of the scale from the original language (English) to the target language (Turkish) were performed by two native Turkish translators. The two translations were compared, and differences that might reflect more ambiguous wording in the original or inconsistencies in the translation process were noted. The two translators synthesized the results of the translations. Based on the versions of the first translator (T1) and the second translator (T2), as well as the original questionnaire, a synthesis of these translations was first made (producing a single translation T-12). The T-12 version of the questionnaire was then back-translated into English by two native English translators who were completely unaware of the original version. Any inconsistencies or conceptual errors between the back-translation and the original source version were checked. Then, all translated forms were examined, and a penultimate version was created to be used in the pretest in line with the consistency between the translated and original forms. The final stage of the cultural adaptation process, the pretest, was conducted. Thirty participants with IA completed the Turkish WALS and then underwent a cognitive debriefing interview about the length, ease, relevance and comprehensiveness of the items. There were no unclear statements. After these interviews, the final version of the scale was determined.

**İŞYERİ AKTİVİTE KISITLILIKLARI ÖLÇEĞİ (İAKÖ)**

**Açıklamalar:** İAKÖ için iki farklı yönerge seti kullanılmaktadır ve ölçeğin uygulanacağı örneklemlere göre değişiklik göstermektedir. İki farklı yönerge kullanıldığında ölçeğin psikometrik özellikleri benzer bulunmuştur.

**Orijinal açıklamalar:** “Bu sorular ile size işinizle ilgili aktiviteler hakkında soru sormak istiyoruz. Bu aktivitelerde ne kadar zorlandığınızı düşündüğünüzde, bunları başka bir kişinin yardımı olmadan veya özel bir alet veya ekipman yardımı olmadan yapmayı düşünmenizi istiyoruz.”

Alternatif açıklamalar kronik rahatsızlıkları olan kişilerde ve sağlıklı kontrollerde kullanılmaktadır:

“Pek çok insan, yaptıkları iş aktivitelerinin türü, işe gidip gelme veya işin programlanması (örneğin, vardiyalı çalışma) nedeniyle işte zorluğa sahiptir. Aşağıdaki sorular için, sahip olabileceğiniz herhangi bir sağlık sorununun işinizde sizin için ek sorun yaratıp yaratmadığını değerlendirmek istiyoruz.

Lütfen sağlığınızla ilgili aşağıdaki işle ilgili aktivitelerde herhangi bir zorluk yaşayıp yaşamadığınızı düşünün.”

|  |  |  |
| --- | --- | --- |
| 1. İşe gidip gelmede (örn. metro, otobüs, araba, yürüyüş) ve işe zamanında gidip gelmekte ne kadar zorluk yaşıyorsunuz? | 0 Zorluk yok  1 Biraz zorluk  2 Çok fazla zorluk  3 Yapamıyorum | 77 Sağlık durumumla ilişkisiz zorluk  88 Reddedildi  99 Uygulanamaz (Örn. evde çalışma) |

|  |  |  |
| --- | --- | --- |
| 1. İşyerinde dolaşmakta ne kadar zorluk yaşıyorsunuz (örn. merdivenler, koridorlar, mobilyalar)? | 0 Zorluk yok  1 Biraz zorluk  2 Çok fazla zorluk  3 Yapamıyorum | 77 Sağlık durumumla ilişkisiz zorluk  88 Reddedildi  99 Uygulanamaz (Örn. evde çalışma) |

|  |  |  |
| --- | --- | --- |
| 1. İşinizde uzun süre (örneğin 20 dakikadan fazla) oturmakta ne kadar zorluk yaşıyorsunuz? | 0 Zorluk yok  1 Biraz zorluk  2 Çok fazla zorluk  3 Yapamıyorum | 77 Sağlık durumumla ilişkisiz zorluk  88 Reddedildi  99 Uygulanamaz (Örn. evde çalışma) |

|  |  |  |
| --- | --- | --- |
| 1. İşinizde uzun süre (örneğin 20 dakikadan fazla) ayakta durmakta ne kadar zorluk yaşıyorsunuz? | 0 Zorluk yok  1 Biraz zorluk  2 Çok fazla zorluk  3 Yapamıyorum | 77 Sağlık durumumla ilişkisiz zorluk  88 Reddedildi  99 Uygulanamaz (Örn. evde çalışma) |

|  |  |  |
| --- | --- | --- |
| 1. Nesneleri kaldırmakta, taşımakta veya hareket ettirmede ne kadar zorluk yaşıyorsunuz? | 0 Zorluk yok  1 Biraz zorluk  2 Çok fazla zorluk  3 Yapamıyorum | 77 Sağlık durumumla ilişkisiz zorluk  88 Reddedildi  99 Uygulanamaz (Örn. evde çalışma) |

|  |  |  |
| --- | --- | --- |
| 1. Ellerinizle çalışırken ne kadar zorluk yaşıyorsunuz (örn. yazı yazmak, klavye kullanmak, küçük nesneleri kavramak, telefonu tutmak)? | 0 Zorluk yok  1 Biraz zorluk  2 Çok fazla zorluk  3 Yapamıyorum | 77 Sağlık durumumla ilişkisiz zorluk  88 Reddedildi  99 Uygulanamaz (Örn. evde çalışma) |

|  |  |  |
| --- | --- | --- |
| 1. Çömelme, eğilme, diz çökme veya değişik pozisyonlarda çalışma konusunda ne kadar zorluk yaşıyorsunuz? | 0 Zorluk yok  1 Biraz zorluk  2 Çok fazla zorluk  3 Yapamıyorum | 77 Sağlık durumumla ilişkisiz zorluk  88 Reddedildi  99 Uygulanamaz (Örn. evde çalışma) |

|  |  |  |
| --- | --- | --- |
| 1. Ulaşmakta ne kadar zorluk yaşıyorsunuz? | 0 Zorluk yok  1 Biraz zorluk  2 Çok fazla zorluk  3 Yapamıyorum | 77 Sağlık durumumla ilişkisiz zorluk  88 Reddedildi  99 Uygulanamaz (Örn. evde çalışma) |

|  |  |  |
| --- | --- | --- |
| 1. İşinizin gerektirdiği program veya çalışma saatleri ile ilgili ne kadar zorluk yaşıyorsunuz? | 0 Zorluk yok  1 Biraz zorluk  2 Çok fazla zorluk  3 Yapamıyorum | 77 Sağlık durumumla ilişkisiz zorluk  88 Reddedildi  99 Uygulanamaz (Örn. evde çalışma) |

|  |  |  |
| --- | --- | --- |
| 1. İşinizin gerektirdiği iş temposunda ne kadar zorlanıyorsunuz? | 0 Zorluk yok  1 Biraz zorluk  2 Çok fazla zorluk  3 Yapamıyorum | 77 Sağlık durumumla ilişkisiz zorluk  88 Reddedildi  99 Uygulanamaz (Örn. evde çalışma) |

|  |  |  |
| --- | --- | --- |
| 1. Konsantre olmakta ya da aklınızı işe vermekte ne kadar zorluk yaşıyorsunuz? | 0 Zorluk yok  1 Biraz zorluk  2 Çok fazla zorluk  3 Yapamıyorum | 77 Sağlık durumumla ilişkisiz zorluk  88 Reddedildi  99 Uygulanamaz (Örn. evde çalışma) |

|  |  |  |
| --- | --- | --- |
| 1. Genel olarak, mevcut iş taleplerinizi karşılamakta ne kadar zorluk yaşıyorsunuz? | 0 Zorluk yok  1 Biraz zorluk  2 Çok fazla zorluk  3 Yapamıyorum | 77 Sağlık durumumla ilişkisiz zorluk  88 Reddedildi  99 Uygulanamaz (Örn. evde çalışma) |

0=zorluk yok

0-4 puan= hafif zorluk

5-8 puan= orta derecede zorluk

9 ve üzeri= önemli derecede işyeri zorluğu
