# Supplementary material for: Cross-cultural adaptation and psychometric testing of the Turkish Version of the Workplace Activity Limitations Scale (WALS) in people with inflammatory arthritis
Source: Rheumatol Adv Pract. 2026 Feb 27;10(1):rkag028. doi: 10.1093/rap/rkag028 (PMC12975718; doi:10.1093/rap/rkag028)
Supplement: rkag028_Supplementary_Data [file rkag028_supplementary_data.zip › Supplementary File 2.docx]

**Supplementary File 2: Content validity of Workplace Activity Limitations Scale linked to the ICF Core Set for Vocational Rehabilitation (Activities and Participation domain).**

| **Work Activity Limitations Scale items** | | | | | | | | | | | | | |
| --- | --- | --- | --- | --- | --- | --- | --- | --- | --- | --- | --- | --- | --- |
| **ICF Vocational Rehabilitation Core Set Activities and Participation domain** | | 1  To/ from work | 2  Get around work | 3  Sit | 4  Stand | 5  Lift,  carry | 6  Work with hands | 7  Crouch, bend, kneel | 8  Reach | 9  Manage hours | 10  Manage pace | 11  Job demands | 12  Concen-tration |
| 1 | d155 Acquiring skills |  |  |  |  |  |  |  |  |  |  |  |  |
| 2 | d160 Focusing attention |  |  |  |  |  |  |  |  |  |  |  |  |
| 3 | d163 Thinking |  |  |  |  |  |  |  |  |  |  |  |  |
| 4 | d166 Reading |  |  |  |  |  |  |  |  |  |  |  |  |
| 5 | d170 Writing |  |  |  |  |  |  |  |  |  |  |  |  |
| 6 | d172 Calculating |  |  |  |  |  |  |  |  |  |  |  |  |
| 7 | d175 Solving problems |  |  |  |  |  |  |  |  |  |  |  |  |
| 8 | d177 Making decisions |  |  |  |  |  |  |  |  |  |  |  |  |
| 9 | d210 Undertaking a single task |  |  |  |  |  |  |  |  |  |  |  |  |
| 10 | d220 Undertaking multiple tasks |  |  |  |  |  |  |  |  |  | XXX |  |  |
| 11 | d230 Carrying out daily routine |  |  |  |  |  |  |  |  |  |  |  |  |
| 12 | d240 Handling stress and other psychological demands |  |  |  |  |  |  |  |  |  |  |  |  |
| 13 | d310 Communicating with - receiving - spoken messages |  |  |  |  |  |  |  |  |  |  |  |  |
| 14 | d315 Communicating with - receiving - nonverbal messages |  |  |  |  |  |  |  |  |  |  |  |  |
| 15 | d350 Conversation |  |  |  |  |  |  |  |  |  |  |  |  |
| 16 | d360 Using communication devices and techniques |  |  |  |  |  |  |  |  |  |  |  |  |
| 17 | d410 Changing basic body position |  |  |  |  |  |  |  |  |  |  |  |  |
| 18 | d415 Maintaining a body position |  |  |  |  |  |  |  |  |  |  |  |  |
| 19 | d430 Lifting and carrying objects |  |  |  |  |  |  |  |  |  |  |  |  |
| 20 | d440 Fine hand use |  |  |  |  |  |  |  |  |  |  |  |  |
| 21 | d445 Hand and arm use |  |  |  |  |  |  |  |  |  |  |  |  |
| 22 | d450 Walking |  |  |  |  |  |  |  |  |  |  |  |  |
| 23 | d455 Moving around |  |  |  |  |  |  |  |  |  |  |  |  |
| 24 | d465 Moving around using equipment |  |  |  |  |  |  |  |  |  |  |  |  |
| 25 | d470 Using transportation |  |  |  |  |  |  |  |  |  |  |  |  |
| 26 | d475 Driving |  |  |  |  |  |  |  |  |  |  |  |  |
| 27 | d530 Toileting |  |  |  |  |  |  |  |  |  |  |  |  |
| 28 | d540 Dressing |  |  |  |  |  |  |  |  |  |  |  |  |
| 29 | d570 Looking after one’s health |  |  |  |  |  |  |  |  |  |  |  |  |
| 30 | d710 Basic interpersonal interactions |  |  |  |  |  |  |  |  |  |  |  |  |
| 31 | d720 Complex interpersonal interactions |  |  |  |  |  |  |  |  |  |  |  |  |
| 32 | d740 Formal relationships |  |  |  |  |  |  |  |  |  |  |  |  |
| 33 | d820 School education |  |  |  |  |  |  |  |  |  |  |  |  |
| 34 | d825 Vocational training |  |  |  |  |  |  |  |  |  |  |  |  |
| 35 | d830 Higher education |  |  |  |  |  |  |  |  |  |  |  |  |
| 36 | d840 Apprenticeship (work preparation |  |  |  |  |  |  |  |  |  |  |  |  |
| 37 | d845 Acquiring, keeping, and terminating a job |  |  |  |  |  |  |  |  |  |  |  |  |
| 38 | d850 Remunerative employment |  |  |  |  |  |  |  |  |  |  |  |  |
| 39 | d855 Non-remunerative employment |  |  |  |  |  |  |  |  |  |  |  |  |
| 40 | d870 Economic self-sufficiency |  |  |  |  |  |  |  |  |  |  |  |  |

Note: Item 11: “Meeting your current job demands” predominantly relates to Chapter 2: General Tasks and Demands” in the ICF, which states that “These items can be used in conjunction with more specific tasks or actions to identify the underlying features of the execution of tasks in different circumstances.” Codes shown in light grey may be relevant depending on the nature of the person’s job and how the respondent interprets this WALS item.
