# Supplementary material for: Cross-cultural adaptation and psychometric testing of the Turkish Version of the Workplace Activity Limitations Scale (WALS) in people with inflammatory arthritis
Source: Rheumatol Adv Pract. 2026 Feb 27;10(1):rkag028. doi: 10.1093/rap/rkag028 (PMC12975718; doi:10.1093/rap/rkag028)
Supplement: rkag028_Supplementary_Data [file rkag028_supplementary_data.zip › Supplementary File 3.docx]

**Supplementary File 3:**

**Cross-cultural adaptation and psychometric testing of the Turkish version of the Workplace Activity Limitations Scale (WALS) in people with inflammatory arthritis**

Given the dimensionality problem, and the local dependent items, a super-item solution was derived. This resolved the problem, retaining 97% of the residual non-error variance, and just managing to pass the unidimensionality test with the lower Confidence interval (LCI) being 4.5%. As the original DIF pattern indicated a possible conceptual-based solution, this also found a satisfactory solution when applies as a testlet model, with a more robust conditional Chi-Square test of fit. A transformation from the raw score to the Rasch metric was based upon this solution (Supplementary Table 1).

Testing the invariance of the WALS between the British English and Turkish Adaptations

Data was merged between the original English data set and the recent Turkish adaptation set. The same testlet based solution was applied to the merged data, that is one representing physical functioning, the other managing the work environment. The latter displayed DIF by country, where there appeared to be slightly less problems with the work environment in the UK than in Turkey at any level of work limitations. The UK also showed slightly more problems with functioning, but not statistically significant. Testing whether or not these differences cancelled out, the environment super item was split to derive a country-specific set, and the analysis re-run. The difference in estimates between the split and unsplit solution (the latter anchored on the function super item from the split solution which was unbiased), gave a t-test value of 0.292. Furthermore, the effect size of the difference between estimates was 0.003, which is considered negligible. Consequently, the WALS can be considered invariant across countries.

***Full Analysis***

Reliability and Precision

Given the WALS showed adequate fit to the Rasch model and was shown to be invariant with the original English version, the WALS metric estimates were exported into the main Turkish data file for further analysis. The metric value of the WALS at baseline was 13.4 (SD6.6). For those who responded for the retest, their baseline value was 14.44 (SD 6.7), whereas their retest value was 14.62 (SD7.2), giving a metric test-retest correlation reliability of 0.90. This gave a Standard Error of Measurement (SEM) of 2.09, and a Smallest Detectable Difference (SDD) of 5.8, or 16.1% of the operational range of the scale.

Supplementary Table 1. Raw-Score to Metric Transformation

| Raw Score | Metric |
| --- | --- |
| 0 | 0.0 |
| 1 | 2.0 |
| 2 | 3.5 |
| 3 | 4.6 |
| 4 | 5.5 |
| 5 | 6.4 |
| 6 | 7.2 |
| 7 | 8.1 |
| 8 | 9.1 |
| 9 | 10.0 |
| 10 | 11.1 |
| 11 | 12.1 |
| 12 | 13.3 |
| 13 | 14.5 |
| 14 | 15.9 |
| 15 | 17.3 |
| 16 | 18.8 |
| 17 | 20.2 |
| 18 | 21.7 |
| 19 | 23.2 |
| 20 | 24.6 |
| 21 | 26.0 |
| 22 | 27.1 |
| 23 | 28.0 |
| 24 | 28.7 |
| 25 | 29.3 |
| 26 | 29.8 |
| 27 | 30.2 |
| 28 | 30.7 |
| 29 | 31.1 |
| 30 | 31.6 |
| 31 | 32.0 |
| 32 | 32.5 |
| 33 | 33.1 |
| 34 | 33.8 |
| 35 | 34.7 |
| 36 | 36.0 |
